# Simultaneous Hydrolysis of Ellagitannins and Extraction of Ellagic Acid from Defatted Raspberry Seeds Using Natural Deep Eutectic Solvents (NADES)

**DOI:** 10.3390/antiox11020254

**Published:** 2022-01-28

**Authors:** Nemanja Teslić, Filipa Santos, Filipe Oliveira, Alena Stupar, Milica Pojić, Anamarija Mandić, Branimir Pavlić, Aleksandra Cvetanović Kljakić, Ana Rita C. Duarte, Alexandre Paiva, Aleksandra Mišan

**Affiliations:** 1 Institute of Food Technology, University of Novi Sad, Blvd. cara Lazara 1, 21000 Novi Sad, Serbia; alena.tomsik@fins.uns.ac.rs (A.S.); milica.pojic@fins.uns.ac.rs (M.P.); anamarija.mandic@fins.uns.ac.rs (A.M.); aleksandra.misan@fins.uns.ac.rs (A.M.); 2 LAQV, REQUIMTE, Departamento de Química, Nova School of Science and Technology, 2829-516 Caparica, Portugal; mfca.santos@campus.fct.unl.pt (F.S.); fsn.oliveira@campus.fct.unl.pt (F.O.); ard08968@fct.unl.pt (A.R.C.D.); abp08838@fct.unl.pt (A.P.); 3 Faculty of Technology, University of Novi Sad, Blvd. cara Lazara 1, 21000 Novi Sad, Serbia; bpavlic@uns.ac.rs (B.P.); a.c.istrazivac@gmail.com (A.C.K.)

**Keywords:** ellagic acid, hydrolysis and extraction, by-product valorization, acidic NADES

## Abstract

Defatted raspberry seeds were used as an alternative source of antioxidants and ellagic acid (EA) extracted using Natural deep eutectic solvents (NADES). In the preliminary study, the best NADES combination (citric acid-betaine) and the most influential variables (temperature, time, and NADES/plant ratio) were selected for the further optimization process. All samples were analyzed in terms of total polyphenol, EA content, and antioxidant activity. Two sets of optimal conditions were generated by response surface methodology. The first set (Opt1) was designed for higher conversion of ellagitannins to EA while the latter set (Opt2) for higher EA content/100 g extract. Opt1 and Opt2 had higher values for all investigated responses compared to 80% ethanolic extract but had a lower conversion rate of ellagitannins to EA compared to acidified methanol extract. The third set of parameters (Opt3) selected beyond the initial experimental domain was used to obtain a sample with the highest EA content/100 g extract. Due to their nature, NADES extracts are ready to use and could have various technological roles in products since they are antioxidants, acidifiers, and colorants. NADES raspberry extracts exhibited higher anti-proliferative activity compared to ethanolic extracts in terms of EC_50_ values. However, the main contributor of anti-cancer activity in NADES raspberry extracts were individual NADES compounds and/or their newly formed NADES structure.

## 1. Introduction

In 2019, Serbia was the third-largest raspberry (*Rubus idaeus* L.) producer in the world, with total annual fruit production of 1.20 × 10^5^ tons [1]. The processing of fresh raspberries during the production of jams, fruit pulp, juices, and other similar products generates a large number of seeds, which are currently underutilized in Serbia. The major constituents of raspberry seeds (RS) are dietary fibers (65.79 g/100 g), oil (14.90 g/100 g), and proteins (6.95 g/100 g) [2]. RS are also a rich source of secondary plant metabolites such as polyphenols which are present in free, soluble bond (esterified or glycosylated), and insoluble-bond forms [3]. One of the most important polyphenols from RS is ellagic acid (EA) due to its broad spectrum of biological effects such as antioxidant, anti-carcinogenic, anti-obesity, anti-inflammatory and anti-angiogenic, anti-neurodegenerative, hepatoprotective activity, although mainly evaluated in vitro [4,5]. The wide range of EA bioactivities is mainly attributed to its ability to scavenge free radicals in tissues, thus decreasing or preventing oxidative stress, which triggers various diseases [4]. The majority of EA in RS is bonded in the form of ellagitannins (EGTs; e.g., sanguiin and lambertianin) which account for up to 1.2 g/100 g in seed weight [5]. EGTs are poorly absorbed in the gastrointestinal tract (GIT) and hydrolyzed predominantly to EA [6]. It is noteworthy that even EA itself has low bioavailability due to its poor water-solubility, metabolism to urolithins in GIT, and the first pass effect [4]. However, increasing the concentration of free EA in food products could, at least potentially, shorten the metabolism path from EGTs to urolithins and/or, if not present in other tissues, could potentially serve as a therapeutic agent for GIT cancers [7,8].

Conventional hydrolysis of EGTs is achieved using acids or alkali in a heated medium. The hydrolysis process could be achieved with emerging technologies, including microwaves, resulting in a higher conversion rate of EGTs to EA, lower reaction time, and less chemical consumption compared to traditional acidic hydrolysis [9]. However, hydrolysis of EGTs to EA, even with this novel technology, would require a certain amount of chemicals harmful to human health. Since natural deep eutectic solvents (NADES) are generated from renewable compounds predominantly present in nature [10], they could serve as a less toxic alternative reaction medium for hydrolysis and sequel extraction of EA. The formation of NADES is induced by molecular charge relocation between hydrogen bond donors (HBD) and acceptors (HBA), resulting in a significant melting point depression of NADES compounds [10] and creating a viscous liquid-like medium. Edible organic acids (citric, malic, tartaric, and lactic acid) could be used as HBD for the purpose of creating favorable acidic conditions for EGTs hydrolysis. Indeed, NADES formulated from organic acids (lactic, malic, and citric acid) and chlorine chloride were already utilized to produce nanocrystalline cellulose from empty fruit bunches [11]. Furthermore, NADES made of oxalic acid and choline chloride could be used as a hydrolysis medium to obtain biofuels and valuable chemicals from lignocellulosic biomass [12]. Thus, it is reasonable to expect that NADES with organic acids could contribute to the hydrolysis of EGTs to EA and increase the quantity of the desired free EA form in the extracts. Betaine is a zwitterionic quaternary ammonium compound that is considered a suitable ingredient for food products in the EU and USA [13] and was already extensively used for NADES preparation as HBA [10]. Furthermore, betaine has several health benefits such as preventing or reducing fat accumulation in the liver, organic osmolyte, and as a regulator of homocysteine in serum, which in elevated concentration leads to homocysteinemia [14]. Thus, using extracts obtained with NADES containing betaine could be used to create functional food products.

Hydrolysis of EGTs to EA and its sequel extraction from RS depends on various processing parameters. Therefore, it is necessary to first screen the most influential parameters and determine the appropriate experimental domain. Particularly since performing optimization with full factorial experimental design with a larger number of variables (5, 6, or more) would require a large number of experimental runs (2^5^ = 32; 2^6^ = 64) which is costly and time-consuming. In that regard, 2 levels of fractional factorial developed by Montgomery [15] could be utilized to screen the most influential variables. The approach of reducing the number of process parameters prior to the final optimization step was already extensively used in other studies [16,17,18]. One of the relevant process parameters is thermal energy which is required for EGTs cleaving [9] but also quicker diffusion of target compounds to extraction medium [19]. Higher temperatures also decrease the viscosity of NADES, which is important since NADES are generally viscous systems at room temperature [19] and have a low mass-transfer rate. Thus, a proper process optimization accounting for all relevant process parameters would be required prior to any potential industrial application.

To meet the world’s increasing demand in nutraceuticals and pharmaceuticals, it is required to use underutilized fruit industry by-products as a cheap raw material to recover valuable bioactives. Also, it is necessary to develop new and sustainable extraction processes which will have a smaller impact on the environment by avoiding the usage of toxic organic solvents. Thus, the presented paper aims to meet such needs by extracting EA from raspberry seeds using a novel extraction method with acidic NADES. To achieve that, it is required to perform four steps: (I) defining the most suitable NADES (organic acids as HBD and betaine or sugars as HBA) for hydrolysis/extraction of EA from RS and comparison with “conventional methods”; (II) determination of the most influential process parameters (e.g., temperature, molar ratio, and time); (III) hydrolysis/extraction optimization for EA recovery from RS using Box-Behnken design coupled with response surface methodology; (IV) determination of the anti-proliferative activity of extracts obtained at optimal conditions.

## 2. Materials and Methods

### 2.1. Chemicals

Anhydrous betaine (>97%) was purchased from Tokyo Chemical Industry (Tokyo, Japan). L(+)-tartaric acid (>99.5%), DL-malic acid (>99.5%) were supplied by Scharlab (Sentmenat, Spain). Citric acid monohydrate (>99.5%) was purchased from AppliChem GmbH (Darmstadt, Germany). Hydrochloric acid was purchased from Lach-Ner (Brno, Czech Republic). Methanol was purchased from Macron (Avantor, Gliwice, Poland). Dimethyl sulfoxide-d_6_ was obtained from Euriso-Top (St. Aubin Cedex, France). Lactic acid (natural, ≥85%) and all other chemicals were obtained from Sigma Aldrich (St. Louis, MO, USA). Human colorectal adenocarcinoma cells (Caco-2, ACC 169) and human caucasian colon adenocarcinoma (HT29, ACC 299) were purchased from DSMZ (Braunschweig, Germany). Roswell Park Memorial Institute (RPMI) medium, fetal bovine serum (FBS), and penicillin–streptomycin (PS) solution were purchased from Corning (Corning, NY, USA). CellTiter 96^®^ AQueous One Solution Cell Proliferation Assay was purchased from Promega (Madison, WI, USA).

### 2.2. Plant Material

Raspberry seeds (cultivar Willamette) which are a by-product from fruit processing was kindly provided by Mondi Lamex (Kraljevo, Serbia). Upon arrival in the laboratory, seeds were dried in a laboratory drier (Sterimaric ST-11, Instrumentaria, Zagreb, Croatia) at 40 °C for 1/2 day. RS water content after drying was 3.25 ± 0.16% which was determined by a laboratory moisture analyzer (MB45, Ohaus, Parsippany, NJ, USA). Dry seeds were milled and then sifted through a sieve (MLU-300, Bühler, Uzwil, Switzerland) to obtain a fraction of 200–400 µm, which was the most suitable for complete defatting process [20]. Supercritical extraction (SFE) with CO_2_ as solvent was applied to obtain defatted raspberry seeds (DRS). Process parameters were set as follows. Pressure: 340 bar, temperature: 51 °C, CO_2_ flow rate: 0.4 kg/h, and extraction time: 4 h. These optimal conditions were determined in a previous study [21]. SFE plant features were described elsewhere [20]. DRS were further used for the extraction of ellagic acid (EA).

### 2.3. NADES Preparation

All NADES used in the present study are composed of organic acids as hydrogen-bond donors and betaine or sugars as hydrogen-bond acceptors with different molar ratios (Table 1 and Table 2). In total, 11 different NADES were selected: lactic acid:fructose:water (LA:FRC:H_2_O; 5:1:0.8), lactic acid:glucose:water (LA:GLC:H_2_O; 5:1:0.8), malic acid:betaine (MA:BET; 1:1 and 2:1), tartaric acid:betaine (TAR:BET; 1:1 and 2:1), lactic acid:betaine:water (LA:BET:H_2_O; 1:1:0.16 and 2:1:0.32), citric acid:betaine:water (CA:BET:H_2_O; 1:1:1, 2:1:2 and 3:1:3). The H_2_O in CA:BET:H_2_O, LA:BET:H_2_O, LA:GLC:H_2_O, and LA:FRC:H_2_O is originated from citric acid monohydrate and water already present in lactic acid, which was also taken into consideration. The final water content for all NADES was mathematically calculated (20 or 25%) and accordingly adjusted. NADES from Table 1 had 20% of water content, while NADES from Table 2 were in the range of 20–25% depending on the experimental setup. NADES were prepared in a water bath at 80 °C placed on a magnetic stirrer hot plate. Mixing lasted approx. 10 min until the stable, transparent liquid was formed. All created NADES were stable at room temperature (~15 °C) for more than seven days.

### 2.4. Preliminary Study Design and Extraction Procedures

The preliminary study was separated into two parts. The first part was the determination of the most suitable NADES in terms of total polyphenols content, DPPH radical scavenging ability, and EA content expressed on NADES and plant material, also comparing these NADES with 80% (*w*/*w*) ethanol and 2M HCl in methanol regarding same target responses. Extractions were performed in a water bath at 60 ± 1 °C placed on a magnetic stirrer hot plate (C-MAG HS 7, IKA, Staufen, Germany) with thermocouple (EST-D5, IKA, Staufen, Germany) for temperature regulation. Solvent (NADES, ethanol 80% or 2M HCL in methanol) and DRS (ratio 20 g/g) were placed together with a small magnet into a glass extraction vial. The vial was tightly closed with a cap and immersed into a water bath for 30 min. After extraction and partial hydrolysis, samples were centrifuged (Biofuge 13, Heraeus Sepatech, Hanau, Germany) for 5 min at 10,000 rpm. The supernatant was separated from solid plant residue and stored in a fridge at 4 °C until the analysis. The method reported by Määttä-Riihinen et al. [22] with slight modifications was applied as a theoretical or complete extraction and hydrolysis process of EA from DRS. Briefly, 0.1 g ± 0.001 g of DRS was weighed on an analytical balance and transferred together with 1 mL of 2M HCl in methanol into a glass extraction vial which was then capped. Hydrolysis and extraction were performed for 2 h at 85 °C. The extracts were then quantitatively transferred into laboratory flasks and filled up to 20 mL with 2M HCl in methanol. Afterward, capped flasks with extracts were sonicated in an ultrasonic bath (XUB5, Grant Instruments, Cambridge, UK) for 30 min at 80 °C and then centrifuged for 5 min at 10,000 rpm. The supernatant was separated from solid plant residue and stored in a fridge at 4 °C until the analysis.

In the second part of the preliminary study, the most potent NADES in terms of the aforementioned responses was used for the determination of the three most influential process parameters. For this purpose, 2^5−1^ factorial design with a total of 16 experimental setups was performed with the following independent variables: extraction temperature (60 and 70 °C), extraction time (30 and 60 min), molar ratio of hydrogen-bond donor (citric acid) in NADES (2 and 3), water content in NADES (20% and 25%) and NADES/plant ratio (N/P—10 and 20 g/g) (Table 2). After extraction, samples were treated as samples in the first part of the preliminary study. All obtained extracts in the preliminary study were assessed in terms of investigated responses, and all extraction procedures were performed in triplicate. 

Coding of independent variables was performed according to the following equation:

(1) $$X=\frac{Xi-X0}{\Delta X}$$

where *X* presents the coded value of process parameters (e.g., extraction temperature), *Xi* stands for actual value, *X*0 corresponds to the actual value in the center of the experimental domain, while Δ*X* stands for the increment of *Xi* corresponding to a variation of 1 unit of *X*. The levels of actual and coded values of all extraction parameters are listed in Table 2.

### 2.5. Box–Behnken Design (BBD) Optimization

The three most influential independent variables from the preliminary study were used in the final step of process optimization. In that regard, the BBD coupled with response surface methodology was applied. Extraction temperature, extraction time, and N/P as selected process parameters were varied on 3 levels (65, 75, and 85 °C; 50, 100, and 150 min; 15, 25, and 35 g/g, respectively) and with 3 replicates at central conditions accounted in total 15 experimental runs (Table 3).

Determination of the relationship between evaluated responses and process parameters was achieved through the fitting target responses in the 2nd order polynomial equation:

(2) $$Y=a_{0}+\sum_{i=1}^{3}a_{i}X_{i}+\sum_{i=1}^{3}a_{ii}X_{i}^{2}+\sum_{i=1}^{2}\sum_{i<j}^{3}a_{ij}X_{i}X_{j\;}$$

where *Y* represent evaluated response, *a*_0_ stands for intercept, *ai*, *aii*, and *aij*, corresponds to linear, interactive, and quadratic regression coefficients of the model, respectively. *Xi* and *Xj* are coded values of selected process parameters. Coding was performed according to Equation (1).

All extractions in the optimization step were performed with CA:BET:H_2_O (3:1:3 molar ratio) and 25% of water content as it was superior compared to other NADES combinations in terms of target responses. The equipment used in optimization was the same as in the first part of the preliminary study. All extractions were performed in triplicate.

### 2.6. Extracts Characterization

#### 2.6.1. Total Polyphenol Content (TPC)

TPC in samples was determined using the modified Folin–Ciocalteu (F-C) method originally presented by Singleton and Rossi [23]. In short, 50 µL of properly diluted sample in aqueous ethanol solution (80% *w*/*w*) was further diluted in a glass tube with 3.95 mL of distilled water. Successively, 0.25 mL of 33.33% aqueous solution F-C reagent (*v*/*v*) and 20% aqueous solution of Na_2_CO_3_ (*w*/*w*) were added in a same tube. Autozero was adjusted with blank prepared with 50 µL of distilled water (including other reagents and pure NADES) instead of DRS extract. Gallic acid was used as the standard for instrument calibration (*R*^2^ = 0.9991). The absorbance reading for all samples and set of gallic acid solutions with different concentrations were measured with UV-VIS spectrophotometer (UV-1800, Shimadzu, Duisburg, Germany) at 750 nm after 60 min incubation period at room conditions. TPC was reported as mg of gallic acid equivalents (GAE) per 100 g dry weight of DRS. All extracts were analyzed in three replicates.

#### 2.6.2. Free DPPH^•^ Scavenging Activity Assay

The method measuring sample ability to scavenge free DPPH radical originally presented by Brand–Williams et al. [24] was conducted according to a modified procedure. Briefly, 0.1 mL of diluted sample was mixed with 2.9 mL of DPPH radical solution in methanol (~25 mg/L). Autozero on a spectrophotometer (UV-1800, Shimadzu, Duisburg, Germany) was adjusted with methanol. The absorbance of DPPH reagent at 517 nm was adjusted to a value of 0.7 ± 0.02. Instrument calibration was performed with a set of Trolox solutions with different concentrations (*R*^2^ = 0.9988). All absorbance readings were performed after a 60 min incubation period in the dark at room temperature. The obtained results were expressed as µM of Trolox equivalents (TE) per g dry weight of DRS. The measurements were conducted in three replicates.

#### 2.6.3. Determination of Ellagic Acid Content by HPLC/DAD

The concentration of ellagic acid (EA) in obtained samples was determined with a slightly modified method described elsewhere [2]. Briefly, before injection into the HPLC system, all extracts were adequately diluted with 80% ethanol (*w*/*w*) and filtered through a 0.45 µm membrane filter. Content of EA in diluted extracts was analyzed using Agilent 1260 series HPLC-DAD system (Agilent Technologies, Santa Clara, CA, USA) and reverse phase Zorbax Eclipse XDB-C18 column (4.6 mm × 50 mm, 1.8 μm particles; Agilent Technologies, Santa Clara, CA, USA). The injection volume was 5 μL, and the column was thermostated at 30 °C. Mobile phase A was pure acetonitrile, while mobile phase B was a 1% aqueous solution of formic acid. Gradient elution program was set as follows: 0–6 min, 85% of B; 6–28 min, 85–50% of B with the flow rate set at 0.5 mL/min. The post-run was set to 5 min. DAD scanning was set at 254 nm, which is the wavelength with maximal UV absorbance for EA, thus used for identification and quantification. The quantity of EA was calculated using a calibration curve (*R*^2^ = 0.9995) and expressed as mg/100 g dry weight of DRS and mg/100 g extract. Analyses were conducted in triplicates for each sample.

#### 2.6.4. Nuclear Magnetic Resonance (NMR)

NMR spectroscopy was performed to obtain 1D and 2D spectra (^1^H-NMR and ^1^H-^1^H NOESY) on a Bruker Avance III 400 spectrometer (Bruker, Billerica, MA, USA) at an operating frequency of 400.13 MHz according to Meneses et al. [25] method. The samples were prepared in a 5 mm NMR tube. For samples with NADES, 350 μL of NADES and 200 μL of DMSO-d_6_ were added, and for pure components, approximately 5 mg of the compound in the NMR tube and 500 μL of DMSO-d_6_ were added. Chemical shifts were referenced to Me4Si (δ in ppm), and the data analysis was performed with MestReNova software (11.0.4-18998).

#### 2.6.5. Anti-Proliferative Activity and Cytotoxicity

The anti-cancer potential of acidic NADES and raspberry extracts was determined in terms of their cytotoxicity, anti-proliferative effects, and selectivity index, according to methods described in previous work [26]. 

The cytotoxic effect was assessed using a continuous cell line culture of Caco-2. Briefly, the cells were subcultured in RPMI medium, supplemented with 10% heat-inactivated FBS and a 1% PS solution. Caco-2 cells were incubated in a humidified atmosphere at 37 °C with 5% CO_2_. The cytotoxicity assay was determined following ISO/EN 10993 guidelines. The culture cells were planted into 96-well plates at a density of 2 × 10^4^ cells/well and allowed to grow for 7 days with medium renewal every 48 h. After 7 days, cells were incubated with culture media (control) and different concentrations diluted in the culture medium of pure acidic NADES used to obtain optimal extracts, NADES raspberry extracts, and ethanolic raspberry extracts. After 1 day, cells were washed 3 to 4 times with PBS due to the intense color of the extracts. The cell viability was assessed using an aqueous one solution for cell proliferation assay containing MTS (3-(4,5-dimethylthiazol-2-yl)-5-(3-carboxymethoxyphenyl)-2-(4-sulfophenyl)-2H-tetrazolium) as the viability reagent. Shortly, 100 μL of the viability reagent was added to each well in a 1:10 dilution and incubated for 3 h. The absorbance was measured at 490 nm using a microplate reader (VICTOR NivoTM, PerkinElmer, Waltham, MA, USA), and cell viability was expressed in terms of the percentage of live cells relative to the control. Three independent experiments were performed in triplicate. 

The anti-proliferative effect of the extracts and NADES towards cancer cells was assessed using continuous cell culture of HT29. Since these cells form a well-differentiated colorectal adenocarcinoma (CRC), they are suitable as a CRC cell model in 2D and 3D in vitro cultures. The cells were subcultured as described for cytotoxicity assay, with slight differences. For the anti-proliferative assay, HT29 cells were seeded at a density of 1 × 10^5^ cells/well in 96-well culture plates. After 24 h, cells were incubated with culture media (control) and different concentrations of diluted in culture medium of the same acidic NADES and extracts. Cell proliferation was measured after 24 h using the MTS viability reagent, as described above. Three independent experiments were performed in triplicate. 

Furthermore, selectivity indexes were calculated as a ratio between the half-maximal effective concentrations (EC_50_) from the cytotoxic and anti-proliferative profiles.

### 2.7. Statistical Analysis

Statistica 13 Software (Statsoft Inc., Tulsa, OK, USA) was utilized for statistical data evaluation using one-way Analysis of variance (ANOVA) and post-hoc Tukey-test. Differences among samples were considered significant if *p* < 0.05. BBD and multiple linear regression analysis were performed using Design Expert 13 software (Stat-Ease, Minneapolis, MN, USA). Fisher’s test was executed to evaluate model quality parameters (adequacy, correlation coefficients, and model statistical significance). The model adequacy was examined according to the obtained correlation coefficient (*R*^2^), adjusted correlation coefficient (*Adj R*^2^), coefficient of variance (*CV*), and *p*-values of applied models and lack of fit testing. Linear, interactive, and quadratic regression coefficients of targeted responses were statistically significant for 0.01 ≤ *p* < 0.05.

## 3. Results and Discussion

### 3.1. Preliminary Study

The preliminary study was divided into two parts. The goal of the first part was to determine the most suitable natural deep eutectic solvent (NADES) for hydrolysis/extraction of ellagitannins (EGTs) to ellagic acid (EA) and to compare NADES efficiency with “conventional methods” (80% aqueous ethanol and 2M HCl methanol). All obtained extracts were also compared in terms of antioxidant activity and total polyphenol concentration (Table 1).

From all prepared acidic NADES, citric acid:betaine:water (CA:BET:H_2_O) with molar ratio 2:1:2 exhibited the highest concentration of EA (75.17 as mg/100 DW of defatted raspberry seeds—DRS; 3.82 mg/solvent mL) even though NADES like tartaric acid:betaine (TA:BET) and malic acid:betaine (MA:BET) (both with molar ratio 2:1) had the same statistical significance (Table 1). Statistically, the most potent free DPPH scavenging activity was observed for extract isolated with lactic acid:betaine:water (LA:BET:H_2_O) and 2:1:0.32 molar ratio (431.18 μM TE/g DW). The highest amount of total polyphenols with statistical significance was also observed for NADES created from LA:BET:H_2_O but with a molar ratio 1:1:0.16 (39.02 mg GAE/g DW). It seems that the increase of acid molarity in all NADES with BET resulted in higher values of almost all target responses suggesting the relevance of acidity for conversion of EGTs to EA (Table 1). As EA is one of the most valuable phenolic compounds in DRS, NADES with CA and BET were selected for further studies.

Since aqueous ethanol is a “safe” organic solvent, relatively cheap, and has appropriate polarity, it is often considered a conventional extraction medium for recovery polyphenols. All acidic NADES extracts were ~1.5–2 fold superior to ethanolic extract in terms of targeted responses, whereby all samples were obtained under the same experimental conditions (Table 1). This could be due to a higher rate of hydrolysis under favorable acidic conditions with NADES made from organic acids and/or higher extraction efficiency of EA compared to ethanol. A recent study evaluated EA recovery from chestnut shells using 10 different NADES and 50% ethanol as extraction mediums coupled with ultrasound-assisted extraction (UAE) [27]. NADES N-propanol:choline chloride (NProp:ChCl) with 1:1 molar ratio exhibited the highest EA concentration (~4.7 mg/g) comparing to all other extraction mediums—e.g., 50% ethanol ~4.2 mg/g. Interestingly, extract obtained with LA:ChCl (molar ratio 1:1) had a significantly lower concentration of EA (~3.3 mg/g) in comparison to 50% ethanol, indicating that besides acidity, other factors like raw material, NADES components, and applied extraction technique, etc. could have an impact on EA content. Another study reported that usage of acidic NADES (ChCl:MA, ChCl:CA, and ChCl:oxalic acid) under certain conditions resulted in higher anthocyanin yield compared to extractions with NADES created from ChCl and sugars [28]. 

On the other hand, both obtained extracts with 2M HCl (see Section 2.4. for experimental conditions) had a higher concentration of EA (703.28–1031.41 mg/100 g DW; 4.34–20.82 mg/100 g extract) and antioxidant activity (597.10–4128.92 μM TE/g DW) compared to acidic NADES (Table 1). A higher conversion rate of EGTs to EA in the presence of strong mineral acid (2 mol of HCl) compared to weak organic acids (1–5 mol of LA and 1–2 mol of TA, CA, and MA) could be a possible explanation for such outcome. Extract recovered with 2M HCl obtained under the same set of experimental conditions as NADES had lower EA concentration (expressed as mg/100 DW) in comparison to extract obtained with 2MHCl but under higher temperature and longer hydrolysis/extraction time (method with theoretical or complete hydrolysis process). This result indicates that other process parameters apart from the selection of extraction medium have a major impact on target responses (Table 1). 

Determination of the most influential process parameters among temperature, molarity ratio, water content, time, and NADES/plant ratio (N/P) was performed in the second part of the preliminary study. Since a large number of experimental trials would be necessary to fully evaluate the impact of all 5 independent variables, 2^5−1^ factorial design was applied, generating in total 16 different sets of experimental parameters (Table 2). All extracts were assessed in terms of the same responses as in the previous step.

Based on the Bonferroni limit (4.355), the most influential factors for non-selective phenolics recovery using solid/liquid extraction (SLE) were the positive effect of water content, interaction of temperature and time, temperature, and molar ratio as the only negative significant effect (Figure S1a). NP was the only significant variable that had a negative impact on free DPPH radical scavenging activity (Figure S1b). For EA yield expressed on DW of DRS, all relevant process parameters had a positive impact. Similarly to antioxidant activity, NP was the most influential independent variable, followed by temperature, time, the interaction of temperature and time, and water content (Figure S1c). EA yield expressed as mg/100 g extract was also selected as the target variable since it represents an actual concentration of EA in extracts, while EA yield expressed on DRS provides information about EA theoretical recovery from seeds. Yet again, NP had the highest impact of EA yield when expressed per 100 g extract, however negative in this case. Other relevant variables had positive impacts (temperature, time, the interaction of temperature and time, water content), excluding interaction of temperature and N/P (Figure S1d).

Similarly to the first step of the preliminary study, EA yield was considered as the most relevant response, thus selected variables for the final optimization step were temperature (65, 75, and 85 °C), time (50, 100, and 150 min), and N/P (15, 25, and 35 g/g). The temperature at 85 °C and time at 150 min were set as upper limits since Määttä–Riihinen et al. [22] were using these conditions for in our case, theoretical determination of EA content, thus comparison of acidified methanol with acidic NADES and ethanol would be more straightforward. Water content in all experiments for final optimization was set at 25%. A certain amount of water in NADES could decrease viscosity, alter polarity, and increase the solubility of target bioactive molecules leading to higher extraction yield [29]. Furthermore, water is also a reactant in the hydrolysis of EGTs to EA. Somewhat similar water content (30%) was selected as optimal for EA recovery from the chestnut shells using NProp:ChCl coupled with UAE [27]. Since a larger quantity of organic acids in NADES had a positive impact on EA yield (Table 1 and Figure S1), the molar ratio of CA:BET:H_2_O was set to 3:1:3 for all experiments in Box–Behnken design, presuming that higher acid quantity in NADES would further increase hydrolysis rate. Influence of temperature, time, and N/P on hydrolysis of EGTs and sequel EA extraction is thoroughly evaluated in Section 3.2.2.

UAE is commonly used in combination with NADES for the recovery of polyphenols from various plants [10]. Therefore, this emerging technology was also tested in combination with acidic NADES. For this purpose, the 2nd and 15th experimental setups were replicated for UAE (samples 17 and 18, respectively; Table 2). Results were surprising since EA concertation was higher in trials obtained by SLE in comparison to UAE (Table 2). Our hypothesis for such an outcome is that intensive stirring on a magnet stirrer hot plate, which occurred during SLE, resulted in a higher mass-transfer rate compared to the cavitation phenomenon caused by UAE, which is one of the main forces during UAE causing rapture of plant cell walls and easier solvent penetration into plant material resulting in higher extraction yields [30]. Another explanation could be the use of an ultrasonic bath instead of an ultrasonic probe. The latter provides direct contact with plant material and could generate up to 100 fold higher power in comparison to ultrasonic baths, which have indirect contact since the sample and solvent are often placed in a glass container during the extraction process [31].

### 3.2. Box–Behnken Design (BBD) and Response Surface Methodology Optimization

#### 3.2.1. Model Adequacy

Analysis of variance (ANOVA) was applied to check fitting quality for 2nd order polynomial models employed for each targeted response (Table S1). High values for determination coefficients (*R*^2^; 0.9770–0.9967) and adjusted regression coefficient (*Adj R*^2^; 0.9357–0.9906) for all investigated responses are the first indicators of proper model fitting to experimentally obtained values. The coefficient of variance (*CV*) represents the dispersion of results from the mean value, and the *CV* value < 10% is commonly considered acceptable. Variance for all target responses was in the range of 1.91–6.40; thus, relatively low *CV* values *CV* are another indicator of adequate fitting of applied quadratic models (Table S1). All employed models were highly significant as p-values determined by Fisher’s test (F-test) were from <0.0001 for EA expressed on DW DRS to 0.0014 for TP content. Adequate fitting of experimental values with predicted values obtained by quadratic models was also confirmed by insignificant lack of fit (with 95% of statistical significance) for all four responses. Therefore, the dispersion of experimentally obtained values was not derived from the model and represents the measure of pure error.

#### 3.2.2. Effect of Independent Variables on Investigated Responses

As was expected, independent variables had an impact on the extraction and hydrolysis process, but their significance varied for each targeted response. According to ANOVA, linear and quadratic terms of N/P and linear terms of temperature had a significant influence on TP content (Table S1). The contributions of each term (linear, interaction, and quadratic) for all parameters were obtained from their respective sum of squares. Linear (79.86%) and quadratic (11.32%) terms of N/P had the most effect on TP content (Figure S2).

A very low quantity of plant material per solvent volume could lead to reduced extraction efficiency since a small amount of plant tissue is processed in a single batch. Reversely, if a large amount of solid sample is immersed during the extraction (in comparison to the low quantity of sample), it could result in slower dispersion of NADES around the sample due to the larger number of solid particles in the extraction medium [13]. Furthermore, as for every solvent, an excessive amount of solid sample could also lead to NADES saturation with target compounds. In those conditions, the mass-transfer rate would decrease, ending with the process underperforming. In our case, TP content increased with higher N/P (Figure S3b,c; Table 3). Higher NADES to plant ratio increased the concentration gradient between two phases, which resulted in enhanced polyphenol recovery from DRS. Thus, the highest TP content was obtained in experimental run 7 (45.76 ± 0.82 mg GAE/g DW; temperature 85 °C, time 100 min, and N/P 35 g/g) while the lowest was in sample 1 (35.54 ± mg GAE/g DW; temperature 65 °C, time 100 min, and N/P 15 g/g; Table 3). Results also suggested that further increase of N/P would not lead to substantially higher TP content as a curve plateau was observed (Figure S3b,c).

The temperature had a positive impact on TP concentration in extracts (Table S1 and Figure S2). However, the temperature impact on TP was much lower in comparison to N/P. In general, mobility on the molecular level increases with higher temperatures enhancing the diffusion of targeted bioactives from plant tissues towards the extraction medium [19]. Most of the polar NADES (e.g., TAR:BET 1:1, CA:BET:H_2_O 2:1:2) are somewhat viscous at room temperature due to extensive H-bonding between HBA and HBD; thus, higher temperatures also decrease the viscosity (Figure S4), which often results in increased recovery of bioactives. On the other hand, the extraction at a higher temperature (e.g., 85 °C) would require a somewhat larger amount of heat energy compared to the extraction at room temperature. In the case of polyphenols, recovery from DRS increase in temperature caused higher extraction yield (Figure S3a,b).

The strongest free radical scavenging activity was detected in extract 14 (1257.69 ± 32.32 µM TE/g DW; temperature 85 °C, time 100 min, and N/P 15 g/g; Table 3). Antioxidant activity of the obtained extract was mostly determined quadratic (65.99%) and linear term (28.79%) of N/P and somewhat affected by the interaction of temperature and N/P (Figure S2 and Table S1). Contrary to TP content, the highest antioxidant activity was achieved with the lowest N/P, suggesting that compounds with stronger antioxidant potential from DRS have lower solubility (Figure S3d–f). Hence, the diffusion of stronger antioxidant compounds from plant tissue towards the extraction medium would be enhanced when the concentration gradient is higher. Interaction of temperature and N/P had a negative impact on the antioxidant activity of DRS extract (Figure S3e). However, since the highest free radical scavenging is obtained with raspberry extracts made with the lowest N/P, it is possible to conclude that under these conditions, temperature has a positive effect on antioxidant activity (Figure S3e), suggesting that more compounds with high thermal stability and high antioxidant activity are extracted with higher temperature and/or there are more hydrolysis products that have higher DPPH free radical scavenging activity.

EA content expressed on DRS and 100 g extract had more significant variables in comparison to TP content and DPPH free radical scavenging activity (Table S1) and varied from 65.38 to 151.56 mg/100 g DW or from 2.12 to 4.83 when expressed as mg/100 g extract (Table 3). All the linear terms (temperature −34.73%, time −10.51%, and N/P—49.22%) had significant and positive effects except for the negative impact of N/P (49.47%) when EA is expressed as mg/100 g extract (Figure S2), indicating that higher N/P (higher concentration gradient) results in percentage-wise more complete hydrolysis and extraction of EA from DRS (Table 3). On the other hand, the concentration of EA per 100 g extract was higher with lower N/P due to the larger quantity of plant material even though exploitation of raw material was reduced under these conditions (Table 3; Figure S3k,l). Extraction time had a positive impact on EA content, suggesting that hydrolysis and sequel extraction demand prolonged exposure to thermal energy when the temperature was up to 85 °C, thus allowing a longer period for EGTs degradation. Extraction time also had a relevant positive impact in NADES/UAE of EA from the chestnut shells, where 70 min (maximum in the experimental domain) was selected as the optimal value [27]. As it was mentioned, the Määttä–Riihinen et al. method [22] was selected as theoretical, and the authors used 85 °C for hydrolysis; thus, the same temperature was set as the upper limit in our study. However, considering that EA is stable up to ~200 °C [9], citric acid up to ~200 °C [32], and anhydrous betaine up to ~245 °C [33], it is more than obvious that temperatures higher than 85 °C would lead to the higher conversion rate of EGTs to EA (Figure S3g,h; Figure S3j,k), but comparison to theoretical method is more straightforward with the selected experimental domain. Such an outcome is rather expected as high temperatures are required for complete hydrolysis of EGTs to EA [9]. EA content (mg/100 g DW DRS) was positively influenced by interactions temperature/time and temperature/NP while EA expressed as mg/100 g extract was determined by positive interaction of temperature/time and negative interaction between temperature and N/P (Table S1). Positive interaction between temperature/time further supports the conclusion that within the selected experimental domain, conversion of EGTs to EA was incomplete, and higher energy demand and/or extended hydrolysis period would be required for such outcome. All equations of second-order polynomial models for investigated responses with significant variables are summarized in Table 4.

In certain cases, like in the present study, the highest values of two responses are achieved under a different set of experimental conditions (Table 3). Somewhat similar conclusions were reported in the study related to extraction of peppermint polyphenols although using microwave-assisted extraction and ethanol as solvent [34]. Authors reported that a wide range of extraction time (~3–17 min) and liquid/solid ratio (~13–19 mL/g) could be used in a series of optimal process conditions, whereas the certain set of process parameters favors total phenol content but disfavors antioxidant activity determined by ABTS assay. Therefore, several optimal conditions could be applied depending on the favored outcome.

#### 3.2.3. Multi-Response Optimization

Two sets of optimal process parameters were selected using desirability function and maximizing EA content expressed as mg/100 g DW (Opt1) or maximizing EA content expressed as mg/100 g extract (Opt2; Table 5). The first set of parameters mimics the process with a higher conversion rate of EGTs to EA, while the latter is used to obtain samples with more EA per 100 g extract. Since it was concluded that higher temperatures and longer hydrolysis period could result in higher EA content, Opt3 was obtained at temperature: 95 °C (limitation for water bath), time: 240 min, and N/P: 10 g/g (Table 5).

**Figure 1:**
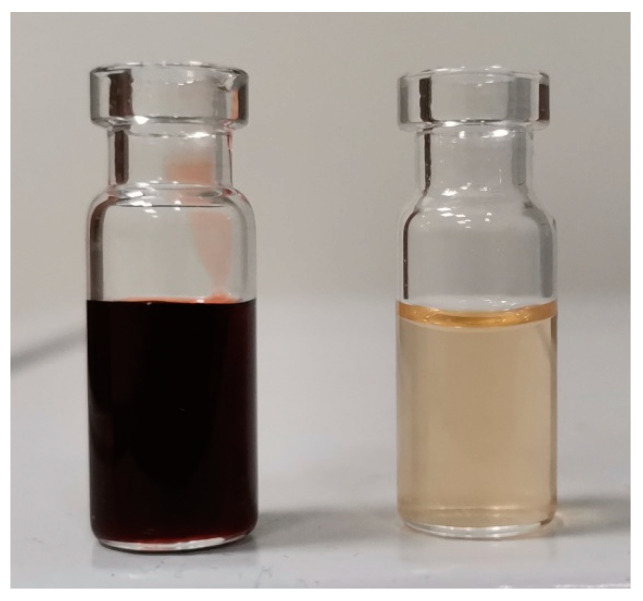
Extract Opt3 (**left**) vs extract ETOH 80% (**right**).

Both sets of optimal conditions (Opt1 and Opt2) obtained with response surface methodology were experimentally validated (Table 5). For sample Opt1 TP content was 44.77 ± 1.76 mg GAE/g DW, antioxidant activity 707.25 ± 2.87 µM TE/g DW, EA content 147.02 ± 7.18 mg/100 g DW, and 2.98 ± 0.15 mg/100 extract. On the other hand, when comparing Opt2 to Opt1, TP content was significantly lower in extract Opt2 (40.16 ± 1.90 mg GAE/g DW), antioxidant activity was significantly higher (995.18 mg µM TE/g DW), while EA content was statistically lower (117.94 ± 5.93 mg/100 g DW) when expressed on DRS and higher when expressed on extract (5.21 ± 0.17 mg/100 extract). Suggesting that indeed the optimal conditions would depend on whether more complete exploitation of plant material is necessary (Opt1) or extract with a higher amount of bioactives per mL is required (Opt2). It is noteworthy that Opt1 and Opt2 had higher values of all selected responses compared to extract obtained with ETOH 80%, indicating that acidic NADES are a good alternative for green solvent, which could be used in food, cosmetic, and pharmaceutic products. In comparison to the theoretical method, samples Opt1 and Opt2 had ~14% and ~11% of EA content when expressed as mg/100 g DW, indicating that the majority of EGTs remained non-susceptible to hydrolysis (Table 5). However, such a process requires the usage of toxic methanol and chlorogenic acid; thus, isolated extracts cannot be used before the intensive purification process. Sample Opt3 obtained at 95 °C during 240 min of extraction had a similar percent of EGTs conversion to EA (~11%) but had the highest concertation of EA within extract (7.93 ± 0.15 mg/100 extract). Indicating that a new study considering higher temperatures (up to 200 °C) and longer extraction time would be necessary before any potential industrial application. Due to their nature, NADES extracts are ready to use and could have various technological roles in products since they are antioxidants (Table 5), acidifiers (citric acid), and colorants (Figure 1).

### 3.3. NMR 

**Figure 2:**
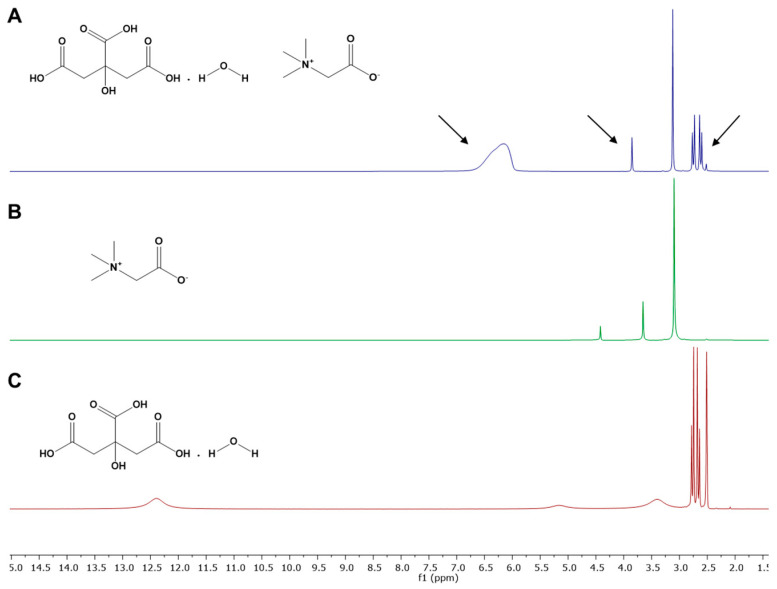
^1^H spectra of (**A**) CA:BET:H_2_O (3:1:3; 25% *w*/*w* of final water); (**B**) betaine; (**C**) citric acid monohydrate.

The NMR experiments were performed in individual components (Figure 2B,C) and in the NADES (CA:BET:H_2_O (3:1:3), Figure 2A) and the ^1^H spectra were overlaid to facilitate the observation of differences between chemical shifts of the individual components and after NADES formation. It is observed that the -OH group of citric acid monohydrate in NADES caused a deviation in the chemical shift from 5.16 to 6.16 ppm. These deviations were not detected when citric acid monohydrate and betaine were submitted to the NMR experiment as individual components (Figure 2B,C). Therefore, results suggest that the interactions between NADES components (betaine and citric acid monohydrate) occur in this group (Figure 2A). The slight deviations on the -CH_2_ groups of betaine and citric acid monohydrate also support the assumption that the interactions occur in the vicinity of these groups. The NMR also shows that 25% *w*/*w* of water in the NADES does not affect the structure of the initial components and contributes to the interactions between the components through hydrogen bonding.

### 3.4. Anti-Proliferative Activity and Cytotoxicity

Colorectal cancer is one of the most lethal and frequently manifested among cancer types, mainly due to the lack of effectiveness of commonly applied anti-cancer therapies. Thus, it is necessary to develop novel and selective therapeutics which are crucial in cancer treatment [35]. As it could be found in literature, naturally occurring EA could be a promising therapeutic compound for cancer treatment [8]. The anti-proliferative activity of EA could be attributed to certain mechanisms on the molecular level, and its activity tends to be cell-specific [8]. Therefore, its anti-cancer potential should be tested for colorectal cancer as well. The results obtained for anti-proliferative activity, cytotoxicity, and consequently selective indexes for optimal NADES raspberry extract (Opt3), raspberry ethanolic extract (ETOH 80%), and acidic NADES are displayed in Table 6.

In the applied methods, HT29 cells represent cancer cells while Caco-2 represent normal intestinal epithelial human cells. All tested samples exhibited higher lethal activity towards HT29 cells compared to Caco-2 cells, highlighted by the selectivity indexes (Table 6). Somewhat high selectivity index was observed for raspberry ethanolic extracts (1.87–5.26) and CA:BET:H_2_O 3:1:3 (3.88), while NADES raspberry extracts exhibited a somewhat lower selectivity index (1.20–2.14), suggesting that all samples exhibited a certain therapeutic window in which cancer cells (HT29) death rate is higher compared to normal intestinal cells (Caco-2). Extracts in ETOH 80% achieved higher EC_50_ compared to Opt3 samples (Table 6), indicating that much a lower concentration of NADES extract can be used for obtaining an anti-proliferative effect in cancer cells. Even though there have been reports on EA of black raspberry seeds having an anti-proliferative effect on HT29 cells [36], when comparing the cell viability results for pure NADES and the NADES raspberry extracts, it seems that the eutectic system itself has a higher selectivity index, thus being cytotoxic towards cancer cells without compromising normal intestinal cells (Caco-2) viability, suggesting that the main contributor to such outcome were individual NADES compounds and/or their newly formed NADES structure. Nevertheless, considering that Opt3 resulted in a selectivity index higher than 2, there is still a certain therapeutic window regarding this NADES extract. The exact mechanism of the EA anti-cancer effect in raspberry extracts could only be hypothesized since only in vitro assays were performed. Depending on the dosage, EA could contribute to the reduction of adenosine triphosphate concentration in cancer cells (Caco-2, MCF-7, Hs 578T, and DU 145) which was associated with a decrease in cell viability [37]. Authors reported that the strong EA anti-cancer effect was confirmed by the disappearance of cellular integrity, nuclear condensation, and DNA fragmentation in cancer cells [37]. EA could also help in the prevention of DNA damage generated by oxidative stress, which could ultimately lead to genetic instabilities in the tumor initiation [38]. The antioxidant mechanism of EA is mainly related to direct scavenging of free radicals, nitrogen reactive species, and oxygen reactive species (ROS), including ^•^OH radicals, ROO^•^ radicals, ^•^NO_2_ radicals, and ONOO− radicals [38]. EA effect on inhibition of ROS production and chelation of metal ions (e.g., copper) could be another mechanism of DNA protection relevant for cancer prevention [38].

There have been several reports on the anti-cancer activity of raspberry extracts. Coates et al. [39] reported that anti-cancer activity for HT29 was also tested with raspberry extract originating from fresh fruit that was treated in a way to mimic digestion in the human body. Several concentrations (0–50 µg/mL GAE) of such extract were submitted to anti-cancer test resulting in a lack of significance in the viability of HT29 cell. The authors also reported that incubation of HT29 for 24 h with the highest extract concentration (50 µg/mL GAE) resulted in only ~10% decrease of HT29 cell viability [39]. Other authors tested in vitro proliferation of human liver cancer cells (HepG2) for four extracts obtained from Heritage, Kiwigold, Goldie, and Anne raspberry varieties [40]. Inhibition of proliferation was determined by dose and raspberry variety. The extract equivalent to 50 mg of Goldie, Heritage, and Kiwigold fruit inhibited the proliferation of HepG2 cells by ~87–89%, while the cultivar Anne exhibited the lowest but still significant inhibition (~70%) of HepG2 cells viability [40]. The literature suggests that obtained results in our study need to be taken with caution and that further testing with in vivo experiments is necessary.

## 4. Conclusions

Underutilized defatted raspberry seeds were used as an alternative source of ellagic acid (EA) and other polyphenols with antioxidant and anti-proliferative properties. Most of EA in raspberry seeds is bound in the form of ellagitannins (EGTs); hence needs to be hydrolyzed. Acidic NADES were successfully prepared and utilized for partial hydrolysis of EGTs to EA and sequel extraction of EA. Samples obtained with two sets of optimal conditions (Opt1 and Opt2) had higher values of all target responses compared to 80% ethanolic extract. However, the same samples had a significantly lower conversion rate of EGTs to EA in comparison to acidified methanolic extract (theoretical method). The NMR studies suggested that the addition of 25% in CA:BET:H_2_O 3:1:3 does not affect the structure of the initial components and contributes to the interactions between the components through hydrogen bonding. According to significantly lower EC_50_ values, NADES raspberry extract exhibited higher anti-proliferative activity compared to ethanolic extract but also had a lower selectivity index. However, the main contributor to anti-cancer activity in NADES raspberry extracts were individual NADES compounds and/or their newly formed NADES structure. Sample Opt3 recovered at temperature: 95 °C, extraction time: 240 min, N/P:10 g/g, molar ratio:3:1:3 and final water content 25% was obtained with conditions beyond experimental limits applied for Box–Behnken design. Opt3 had the highest EA content per 100 g extract, suggesting that a new study with different experimental domains (e.g., temperature up to 200 °C) would be required before any potential commercialization. A molar ratio of 3:1:3 was selected as optimal compared to 1:1:1 and 2:1:2; however, even a higher acid ratio (4:1:4) could potentially increase the EA hydrolysis conversion rate.

## Figures and Tables

**Table 1:** Total polyphenol (TP) content, antioxidant activity, and ellagic acid content obtained by NADES, 80% ethanol solution, and acidified methanol solutions. Extractions were performed at 60 °C for 30 min with 20 g/g of NADES/plant ratio in a water bath placed on a magnetic stirrer hot plate.

Sample	TP [mg GAE ^1^/g DW]	DPPH [μM TE ^2^/g DW ^3^]	Ellagic Acid [mg/100 g DW]	Ellagic Acid [mg/100 g Extract]
LA:FRC:H_2_O (5:1:0.8)	39.80 ± 0.80 ^h^	412.15 ± 26.15 ^c,d^	63.86 ± 2.13 ^a,b^	2.70 ± 0.08 ^b^
LA:GLC:H_2_O (5:1:0.8)	31.72 ± 0.90 ^d^	406.91 ± 6.57 ^c,d^	66.18 ± 1.66 ^a,b^	2.76 ± 0.05 ^b^
MA:BET (1:1)	33.27 ± 1.43 ^d,e^	375.84 ± 8.34 ^b,c^	61.78 ± 3.71 ^a,b^	2.95 ± 0.36 ^b^
MA:BET (2:1)	34.07 ± 0.73 ^d,e,f^	406.36 ± 10.24 ^c,d^	72.01 ± 2.87 ^b^	3.01 ± 0.07 ^b^
TAR:BET (1:1)	26.91 ± 0.64 ^b,c^	348.14 ± 16.60 ^b^	60.13 ± 1.81 ^a,b^	2.59 ± 0.26 ^b^
TAR:BET (2:1)	30.49 ± 1.82 ^c,d^	352.34 ± 27.48 ^b^	71.23 ± 2.11 ^b^	3.18 ± 0.22 ^b^
LA:BET:H_2_O (1:1:0.16)	39.02 ± 1.69 ^g,h^	410.23 ± 9.21 ^c,d^	63.53 ± 1.82 ^a,b^	3.18 ± 0.56 ^b^
LA:BET:H_2_O (2:1:0.32)	35.43 ± 0.61 ^e,f,g^	431.18 ± 6.62 ^d^	63.43 ± 0.98 ^a,b^	3.05 ± 0.38 ^b^
CA:BET:H_2_O (1:1:1)	31.23 ± 2.59 ^d^	387.15 ± 13.46 ^b,c^	66.07 ± 4.31 ^a,b^	2.80 ± 0.15 ^b^
CA:BET:H_2_O (2:1:2)	32.57 ± 1.45 ^d,e^	401.20 ± 8.45 ^c,d^	75.17 ± 3.14 ^b^	3.20 ± 0.13 ^b^
ETOH 80%	20.67 ± 0.73 ^a^	197.78 ± 2.76 ^a^	45.23 ± 0.88 ^a^	1.37 ± 0.03 ^a^
2M HCl in MEOH ^4^	37.33 ± 1.46 ^f,g,h^	4128.92 ± 35.91 ^f^	1031.41 ± 20.73 ^d^	4.34 ± 0.09 ^c^
2M HCl in MEOH	26.54 ± 2.30 ^b^	597.10 ± 8.38 ^e^	703.28 ± 16.25 ^c^	20.82 ± 0.48 ^d^

^1^ Galic acid equivalent; ^2^ Trolox equivalent; ^3^ Dry weight of defatted raspberry seeds; ^4^ Hydrolysis and extraction performed at 85 °C during 2 h in a water bath placed on a magnetic stirrer and sequel sonication in an ultrasonic bath at 80 °C for 30 min. Results are presented as mean values ± sd (*n* = 3) followed by different letters which within the same row represent statistically significant differences (*p* < 0.05) according to post hoc Tukey’s HSD test.

**Table 2:** 2^5−1^ factorial design with process parameters of solid/liquid extraction and experimentally obtained values of total polyphenol (TP) content, antioxidant activity (DPPH), and ellagic acid content. Citric acid monohydrate, betaine (1 mol), and water were used for NADES formulation.

Sample	A: Temperature [°C]		B: Moles of Citric Acid Monohydrate		C: Water Content [g/100 g]		D: Time [min]		E: NADES/Plant Ratio [g/g]		TP [mg GAE ^1^/g DW]	DPPH [μM TE ^2^/g DW ^3^]	Ellagic Acid [mg/100 g DW]	Ellagic Acid [mg/100 g Extract]
Solid/liquid extraction														
1	−1	60	1	3	1	25	1	60	−1	10	34.01 ± 1.05 ^a,b,c,d^	650.43 ± 4.28 ^c,d,e^	45.66 ± 0.24 ^a,b^	3.45 ± 0.09 ^d^
2	1	70	1	3	−1	20	1	60	−1	10	33.25 ± 2.60 ^a,b,c,d^	637.13 ± 12.26 ^c^	52.56 ± 0.03 ^c,d^	3.91 ± 0.01 ^f^
3	1	70	1	3	1	25	1	60	1	20	37.21 ± 0.32 ^e,f^	427.99 ± 9.87 ^a^	72.96 ± 0.90 ^f^	2.74 ± 0.05 ^c^
4	1	70	1	3	1	25	−1	30	−1	10	35.15 ± 1.33 ^b,c,d,e,f^	679.23 ± 10.31 ^d,e^	49.30 ± 0.17 ^b,c^	3.64 ± 0.01 ^e^
5	1	70	−1	2	1	25	−1	30	1	20	36.62 ± 1.41 ^d,e,f^	454.28 ± 11.16 ^a,b^	65.33 ± 0.89 ^e^	2.50 ± 0.04 ^a,b^
6	−1	60	1	3	−1	20	−1	30	−1	10	33.70 ± 0.43 ^a,b,c,d,e^	632.19 ± 11.41 ^c^	45.25 ± 1.79 ^a^	3.39 ± 0.09 ^d^
7	1	70	−1	2	−1	20	1	60	1	20	35.21 ± 0.93 ^b,c,d,e,f^	447.31 ± 2.09 ^a,b^	70.67 ± 0.94 ^f^	2.64 ± 0.02 ^b,c^
8	−1	60	1	3	−1	20	1	60	1	20	31.48 ± 0.80 ^a^	480.17 ± 17.37 ^b^	62.56 ± 0.71 ^e^	2.41 ± 0.01 ^a^
9	−1	60	−1	2	−1	20	1	60	−1	10	32.20 ± 0.87 ^a,b^	643.54 ± 2.12 ^c,d^	45.00 ± 0.19 ^a^	3.42 ± 0.01 ^d^
10	1	70	1	3	−1	20	−1	30	1	20	32.77 ± 0.28 ^a,b,c^	451.86 ± 14.34 ^a,b^	64.18 ± 0.95 ^e^	2.43 ± 0.03 ^a^
11	−1	60	−1	2	1	25	−1	30	−1	10	36.05 ± 0.66 ^c,d,e,f^	655.80 ± 3.56 ^c,d,e^	47.33 ± 2.17 ^a,b^	3.52 ± 0.14 ^d,e^
12	1	70	−1	2	−1	20	−1	30	−1	10	32.74 ± 1.63 ^a,b,c^	641.05 ± 12.33 ^c^	45.35 ± 0.78 ^a,b^	3.43 ± 0.01 ^d^
13	−1	60	−1	2	1	25	1	60	1	20	36.29 ± 0.10 ^c,d,e,f^	455.17 ± 3.03 ^a,b^	69.43 ± 3.87 ^f^	2.56 ± 0.09 ^a,b^
14	−1	60	−1	2	−1	20	−1	30	1	20	33.84 ± 0.44 ^a,b,c,d,e^	436.22 ± 11.59 ^a^	64.71 ± 0.47 ^e^	2.46 ± 0.03 ^a^
15	1	70	−1	2	1	25	1	60	−1	10	38.72 ± 1.90 ^f^	682.23 ± 29.08 ^e^	53.50 ± 0.14 ^d^	3.94 ± 0.03 ^f^
16	−1	60	1	3	1	25	−1	30	1	20	35.08 ± 1.19 ^b,c,d,e^	474.21 ± 14.51 ^b^	65.24 ± 0.29 ^e^	2.45 ± 0.02 ^a^
Additional experiments with ultrasound-assisted extraction														
17	1	70	1	3	−1	20	1	60	−1	10	nd	nd	47.58 ± 0.23	2.06 ± 0.02
18	1	70	−1	2	1	25	1	60	−1	10	nd	nd	51.30 ± 2.58	2.11 ± 0.11

^1^ Galic acid equivalent; ^2^ Trolox equivalent; ^3^ Dry weight of defatted raspberry seeds; Nd—not determined. Results are presented as mean values ± sd (*n* = 3) followed by different letters which within the same row represent statistically significant differences (*p* < 0.05) according to post hoc Tukey’s HSD test.

**Table 3:** Box–Behnken design with process parameters of solid/liquid extraction and experimentally obtained values of total polyphenols (TP) content, antioxidant activity (DPPH), and ellagic acid content expressed on defatted raspberry seeds and NADES extract.

Sample	A: Temperature [°C]		B: Time [min]		C: NADES/Plant Ratio [g/g]		TP [mg GAE ^1^/g DW ^2^]	DPPH [μM TE ^3^/g DW]	Ellagic Acid [mg/100 g DW]	Ellagic Acid [mg/100 g Extract]
1	−1	65	0	100	−1	15	35.54 ± 1.50 ^a^	1051.71 ± 19.65 ^c,d^	65.38 ± 2.15 ^a^	3.19 ± 0.03 ^e,f^
2	−1	65	−1	50	0	25	41.27 ± 0.99 ^c,d^	601.10 ± 16.24 ^a^	85.14 ± 7.83 ^b,c^	2.47 ± 0.15 ^b,c^
3	0	75	1	150	−1	15	37.99 ± 0.92 ^a,b^	1087.24 ± 52.50 ^d^	83.56 ± 3.61 ^b^	4.04 ± 0.13 ^g^
4	1	85	1	150	0	25	43.85 ± 1.23 ^d,e,f^	582.99 ± 21.23 ^a^	143.07 ± 5.00 ^f^	4.02 ± 0.12 ^g^
5	0	75	−1	50	−1	15	36.30 ± 0.85 ^a,b^	1004.22 ± 51.82 ^c^	67.09 ± 1.35 ^a^	3.31 ± 0.04 ^f^
6	1	85	−1	50	0	25	42.04 ± 0.40 ^c,d,e^	634.85 ± 7.96 ^a^	104.16 ± 7.25 ^e^	2.99 ± 0.16 ^d,e^
7	1	85	0	100	1	35	45.76 ± 0.82 ^f^	760.91 ± 30.64 ^b^	151.56 ± 6.04 ^f^	2.97 ± 0.08 ^d,e^
8	0	75	1	150	1	35	43.97 ± 1.90 ^d,e,f^	809.30 ± 20.89 ^b^	137.62 ± 4.39 ^f^	2.68 ± 0.05 ^c,d^
9	0	75	0	50	0	25	43.46 ± 1.11 ^d,e,f^	617.87 ± 29.22 ^a^	105.93 ± 5.39 ^e^	3.03 ± 0.10 ^e,f^
10	−1	65	0	50	1	35	43.38 ± 0.88 ^d,e,f^	820.22 ± 5.32 ^b^	102.97 ± 4.10 ^d,e^	2.12 ± 0.07 ^a^
11	0	75	0	50	0	25	42.61 ± 1.52 ^d,e,f^	597.14 ± 1.43 ^a^	101.09 ± 4.26 ^d,e^	2.96 ± 0.06 ^d,e^
12	−1	65	1	150	0	25	42.59 ± 0.58 ^d,e,f^	621.45 ± 9.81 ^a^	88.06 ± 6.75 ^b,c,d^	2.61 ± 0.12 ^c^
13	0	75	0	100	0	25	43.83 ± 0.89 ^d,e,f^	627.10 ± 7.10 ^a^	101.84 ± 4.99 ^d,e^	2.97 ± 0.10 ^d,e^
14	1	85	0	100	−1	15	38.85 ± 0.73 ^b,c^	1257.69 ± 32.32 ^e^	99.40 ± 4.66 ^c,d,e^	4.83 ± 0.17 ^h^
15	0	75	−1	50	1	35	44.91 ± 0.52 ^e,f^	800.80 ± 5.17 ^b^	109.74 ± 3.62 ^e^	2.22 ± 0.04 ^a,b^

^1^ Galic acid equivalent; ^2^ Trolox equivalent; ^3^ Dry weight of defatted raspberry seeds. Results are presented as mean values ± sd (*n* = 3) followed by different letters which within the same row represent statistically significant differences (*p* < 0.05) according to post hoc Tukey’s HSD test.

**Table 4:** 2nd order polynomial equations only with statistically significant coefficients (*p* < 0.05).

Response	Equation
TP	$Y1=43.30+0.96X_{1}+3.67X_{3}-2.03X_{3}^{2}$
DPPH	$Y2=614.03-151.20X_{3}-66.32X_{1}X_{3}+336.95X_{3}^{2}$
Ellagic acid	$Y3=102.95+19.58X_{1}+10.77X_{2}+23.31X_{3}+9.00X_{1}X_{2}+3.64X_{1}X_{3}+3.74X_{1}^{2}$
Ellagic acid extract	$Y4=2.99+0.55X_{1}+0.30X_{2}-0.67X_{3}+0.22X_{1}X_{2}-0.20X_{1}X_{3}+0.17X_{3}^{2}$

**Table 5:** Experimental validation of optimal conditions obtained with response surface methodology.

Sample	A: Temperature [°C]		B: Time [min]		C: Solvent/Plant Ratio [g/g]		TP [mg GAE ^1^/g DW ^2^]	DPPH [μM TE ^3^/g DW]	Ellagic Acid [mg/100 g DW]	Ellagic Acid [mg/100 g Extract]
Predicted responses										
Opt1-pred	1	85	0.01	100.45	1	35	45.28	772.59	151.56	2.96
Opt2-pred	1	85	0.94	147.00	−0.92	15.76	39.69	1125.64	114.48	5.07
Experimentally obtained responses										
Opt1	1	85	0.01	100.45	1	35	44.77 ± 1.76 ^c^	707.25 ± 2.87 ^b^	147.02 ± 7.18 ^c^	2.98 ± 0.13 ^b^
Opt2	1	85	0.94	147.00	−0.92	15.76	40.16 ± 1.90 ^b^	995.18 ± 32.08 ^c^	117.94 ± 5.93 ^b^	5.21 ± 0.17 ^d^
Opt3 *		95		240		10	37.97 ± 0.60 ^b^	679.97 ± 27.45 ^b^	115.62 ± 4.41 ^b^	7.93 ± 0.15 ^e^
ETOH 80%		60		60		20	20.67 ± 0.73 ^a^	197.78 ± 2.76 ^a^	45.23 ± 0.88 ^a^	1.37 ± 0.03 ^a^
2M HCl in MEOH		85		150		170	37.33 ± 1.46 ^b^	4128 ± 35.91 ^d^	1031.41 ± 20.73 ^d^	4.34 ± 0.09 ^c^

^1^ Galic acid equivalent; ^2^ Trolox equivalent; ^3^ Dry weight of defatted raspberry seeds; Nd—not determined. Experimental results are presented as mean values ± sd (*n* = 3) followed by different letters which within the same row represent statistically significant differences (*p* < 0.05) according to post hoc Tukey’s HSD test. * NADES extract obtained with a set of conditions out of experimental domain in optimization step.

**Table 6:** Anti-proliferative activity (HT29), cytotoxicity (Caco-2), and selectivity index of optimal NADES raspberry extract (Opt3), raspberry ethanolic extract (ETOH 80%), and pure acidic NADES (CA:BET:H_2_O 3:1:3).

Systems/Compounds	EC_50_ (mg/mL)		Selectivity Index
	Cytotoxicity (Caco-2)	Anti-proliferative Activity (HT29)	
Opt3—1 ^a^	3.80 ± 3.33	3.18 ± 0.37	1.20
Opt3—2 ^a^	≈4.63 *	2.16 ± 0.49	2.14
Opt3—3 ^a^	4.60 ± 2.80	≈3.84 *	1.20
ETOH 80%—1 ^b^	103.10 ± 7.04	48.65 ± 11.54	2.12
ETOH 80%—2 ^b^	≈120.60 *	22.93 ± 4.41	5.26
ETOH 80%—3 ^b^	115.30 ± 12.25	61.82 ± 3.17	1.87
CA:BET:H_2_O (3:1:3)	8.06 ± 4.26	2.08 ± 0.14	3.88

* In the range of concentrations tested, it was impossible to accurately determine the SD of the EC50. Other results are expressed as mean ± sd *n* = 3; ^a^—All samples were obtained under the same extraction conditions; ^b^—All samples were obtained under the same extraction conditions.

## Data Availability

Data is contained within the article or supplementary material.

## Supplementary Materials

The following supporting information can be downloaded at: https://www.mdpi.com/article/10.3390/antiox11020254/s1, Figure S1: Pareto chart with effects of process parameters on ellagic acid recovery from defatted raspberry seeds. A-A, B-B, C-C, D-D, E-E stand for individual contribution of temperature, molar ratio, water content, time, and NADES/plant ratio on the process, respectively. AE stands for simultaneous contribution or interaction effect of temperature and NADES/plant ratio on the process and so forth; Figure S2. The contribution of temperature (A), time (B), NADES/plant ratio (C) and their interactions on total polyphenols (TP) content, antioxidant activity (DPPH), and ellagic acid content expressed on defatted raspberry seeds (EA) and NADES extract (EAE); Figure S3. Response surface 3D plots displaying the mutual impact of (a) temperature (T) and time (t); (b) T and NADES/plant ratio (N/P); (c) t and N/P impact on total polyphenol content; (d) T and t; (e) T and N/P; (f) t and N/P effect on antioxidant activity; (g) T and t; (h) T and N/P; (i) t and N/P influence on ellagic acid (EA) expressed on raspberry seeds; (j) T and t; (k) T and N/P; (l) t and N/P effect on EA expressed on extract; Figure S4. Viscosity of NADES in function of temperature. All NADES have 20% *w*/*w* of final water content excluding CA:BET:H_2_O 3:1:3, which has 25% *w*/*w* of final water content.; Table S1. ANOVA of the fitted 2nd order polynomial models obtained with response surface methodology and applied for investigated responses.
