# Supplementary material for: Simultaneous Hydrolysis of Ellagitannins and Extraction of Ellagic Acid from Defatted Raspberry Seeds Using Natural Deep Eutectic Solvents (NADES)
Source: Antioxidants (Basel). 2022 Jan 28;11(2):254. doi: 10.3390/antiox11020254 (PMC8868079; doi:10.3390/antiox11020254)
Supplement: Supplementary file 1 [file antioxidants-11-00254-s001.zip › antioxidants-1544131-supplementary.pdf]

antioxidants

MDPI

Supplementary material

Simultaneous hydrolysis of ellagitannins and extraction of ellagic acid from defatted raspberry seeds using Natural Deep Eutectic Solvents (NADES)

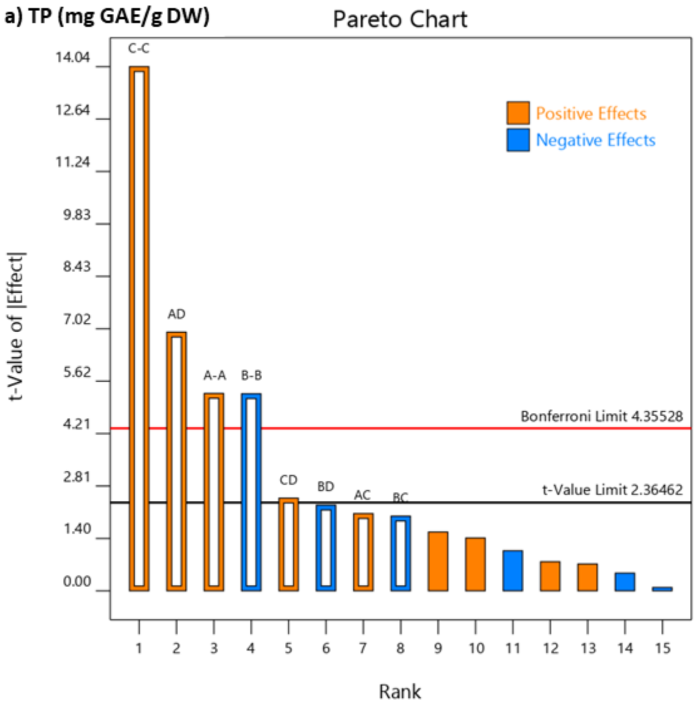

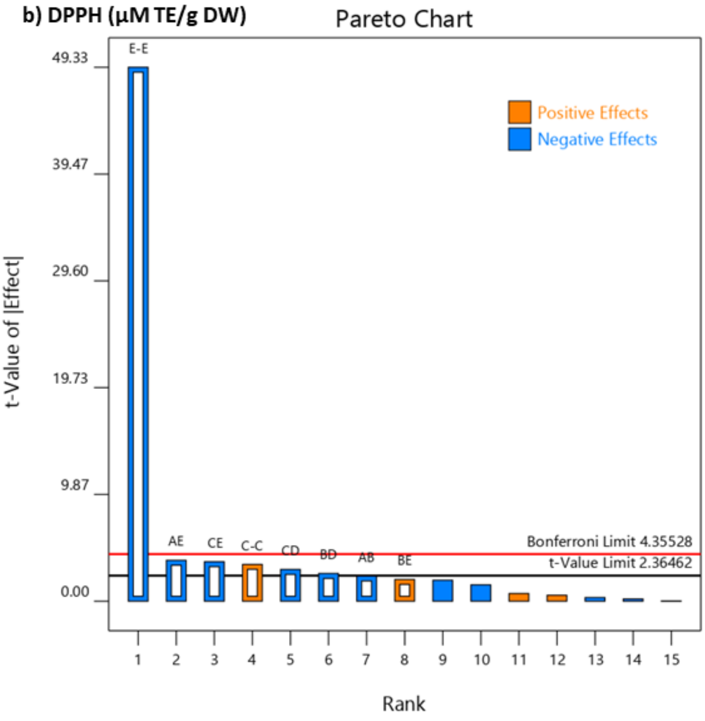

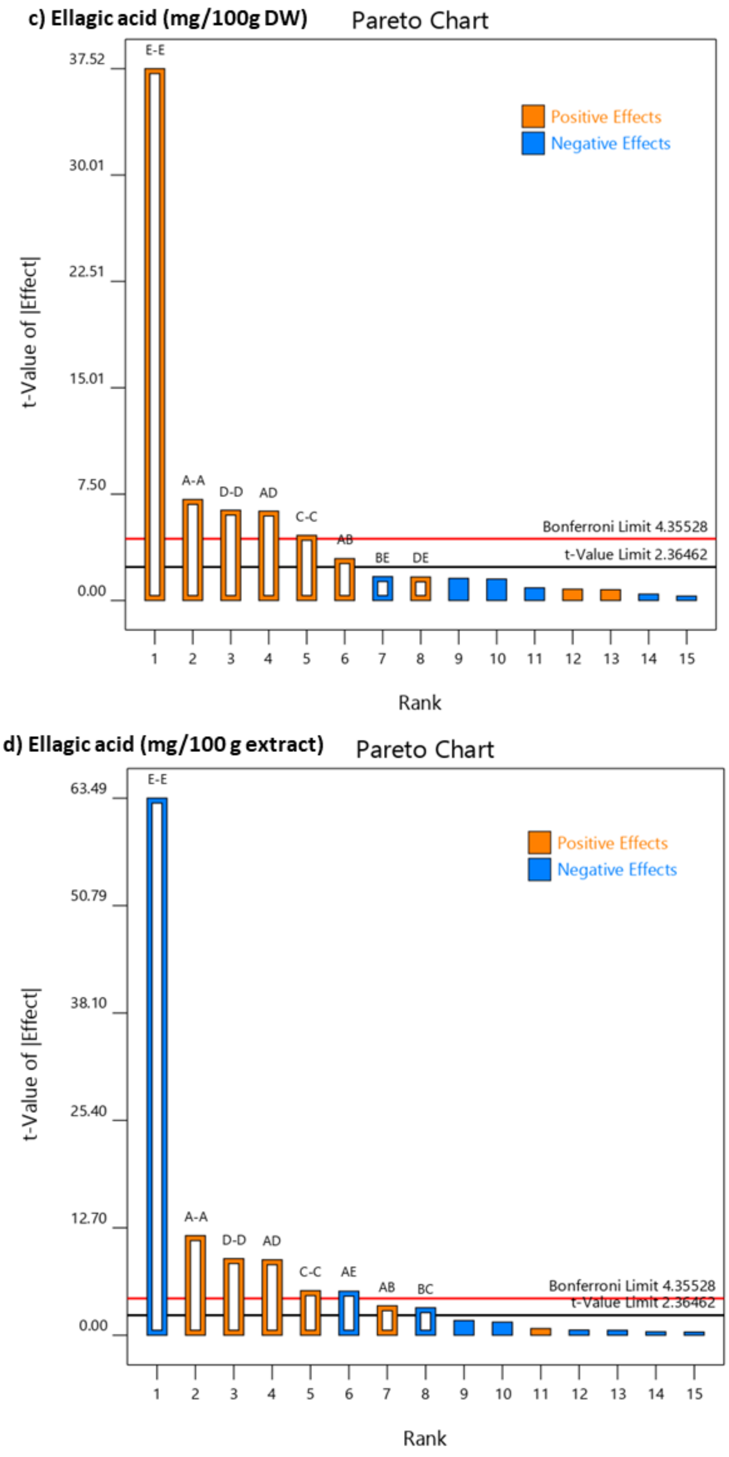

Figure S1. Pareto chart with effects of process parameters on ellagic acid recovery from defatted raspberry seeds. A-A, B-B, C-C, D-D, E-E stand for individual contribution of temperature, molar ratio, water content, time and NADES/plant ratio on process, respectively. AE stands for simultaneous contribution or interaction effect of temperature and NADES/plant ratio on process and so forth.

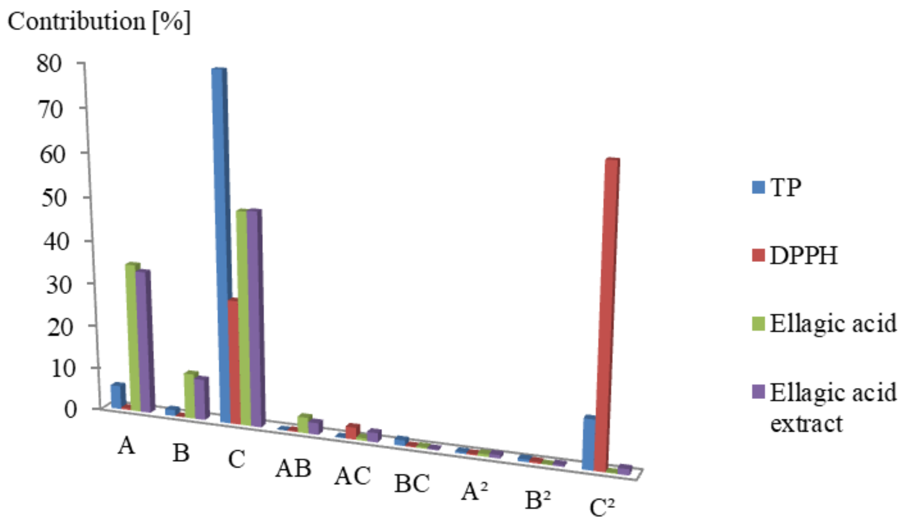

Figure S2. The contribution of temperature (A), time (B), NADES/plant ratio (C) and their interactions on total polyphenols (TP) content, antioxidant activity (DPPH), and ellagic acid content expressed on defatted raspberry seeds (EA) and NADES extract (EAE).

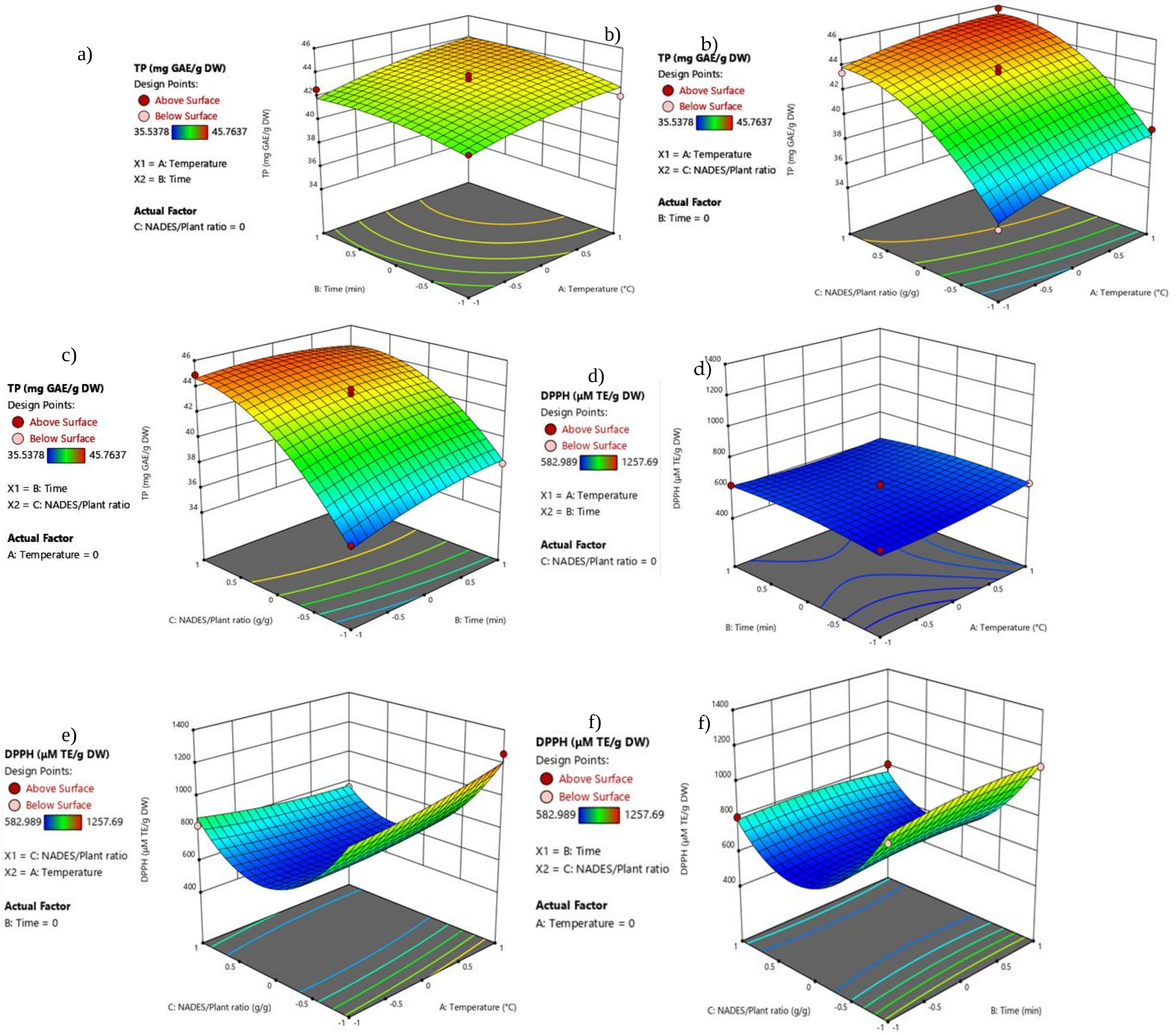

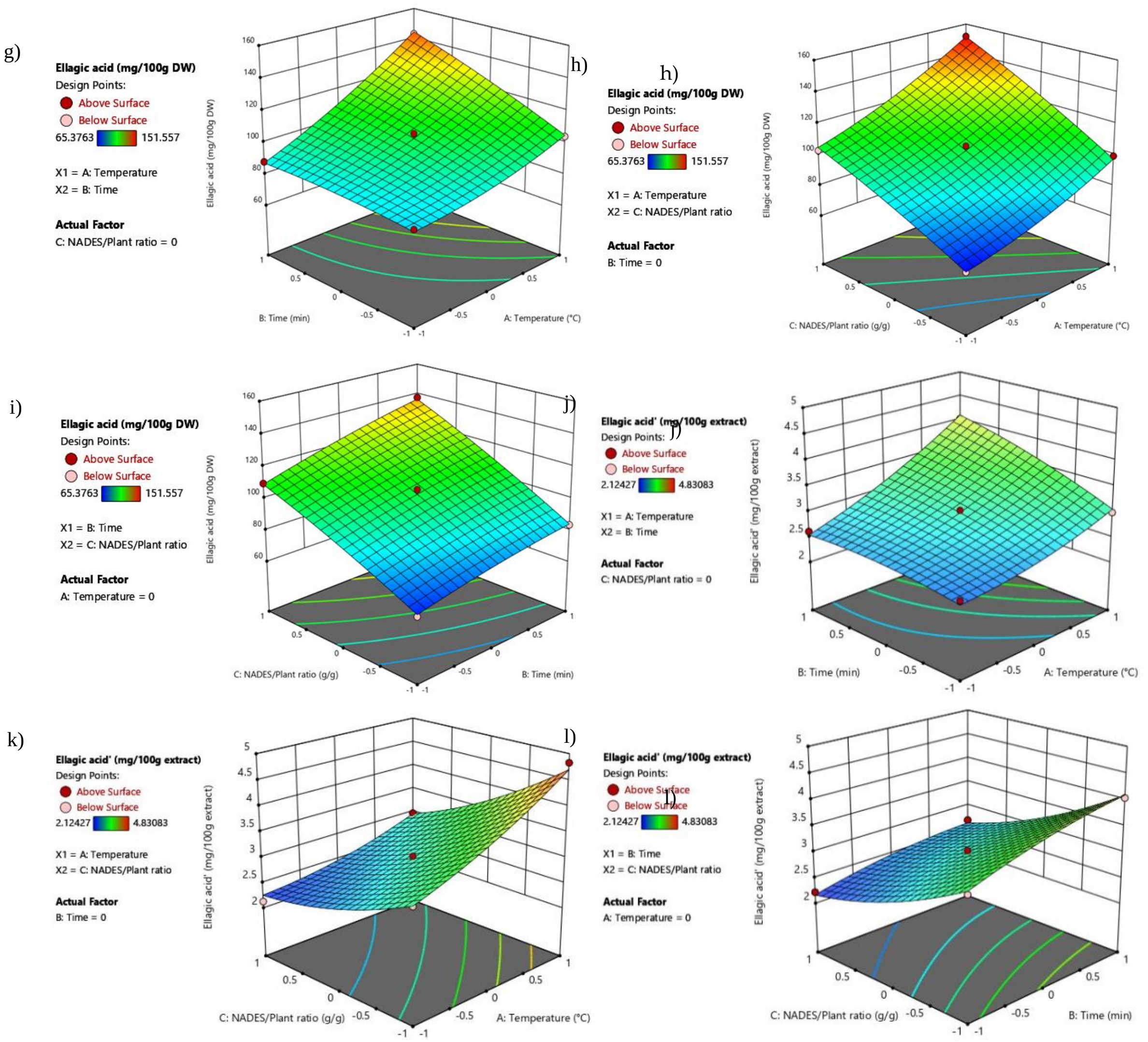

Figure S3. Response surface 3D plots displaying mutual impact of : a) temperature (T) and time (t); b) T and NADES/plant ratio (N/P); c) t and N/P impact on total polyphenol content; d) T and t; e) T and N/P; f) t and N/P effect on antioxidant activity; g) T and t; h) T and N/P; i) t and N/P influence on ellagic acid (EA) expressed on raspberry seeds; j) T and t; k) T and N/P; l) t and N/P effect on EA expressed on extract.

Table S1. ANOVA of the fitted 2nd order polynomial models obtained with response surface methodology and applied for investigated responses.

Term	TP	DPPH	Ellagic acid	Ellagic acid extract
Intercept				
a_0	43.30	614.03	102.95	2.99
Linear				
a_1	0.96**	17.75	19.58*	0.55*
a_2	0.49	7.50	10.77*	0.30*
a_3	3.67*	-151.20*	23.31*	-0.67*
Interaction				
a_{12}	0.12	-18.05	9.00*	0.22**
a_{13}	-0.23	-66.32**	3.64**	-0.20**
a_{23}	-0.66	-18.63	2.85	-0.07
Quadratic				
a_{11}	-0.38	21.65	3.74**	0.12
a_{22}	-0.48	-25.59	-1.59	-0.09

a_{33}	-2.03*	336.95*	-1.87	0.17**
R^{2a}	0.9770	0.9802	0.9967	0.9904
Adj R^{2b}	0.9357	0.9445	0.9906	0.9731
CV^c	1.91	6.40	2.36	3.85
$p_m{}^d$	0.0014*	0.0010*	<0.0001*	0.0002*
$p_{lf}{}^e$	0.3449	0.0545	0.6004	0.0677

*$p<0.01$

**$0.01 \leq p<0.05$

[a] Determination coefficient

[b] Adjusted determination coefficient

[c] Coefficient of variance (%)

[d] Significance of model

[e] Lack of fit

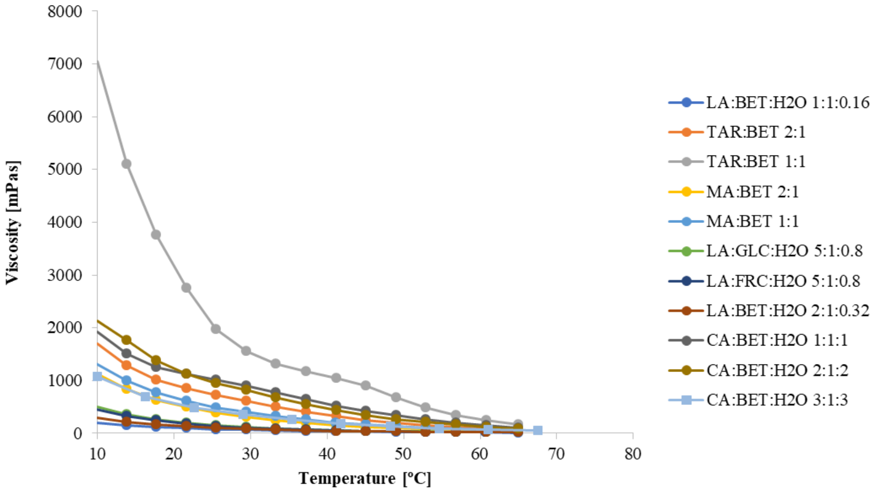

Figure S4. Viscosity of NADES in function of temperature. All NADES have 20% w/w of final water content excluding CA:BET:H_2O 3:1:3 which has 25% w/w of final water content.
